# Protective Function of Novel Fungal Immunomodulatory Proteins Fip-lti1 and Fip-lti2 from *Lentinus tigrinus* in Concanavalin A-Induced Liver Oxidative Injury

**DOI:** 10.1155/2019/3139689

**Published:** 2019-05-06

**Authors:** Yingnyu Gao, Ying Wáng, Ying Wāng, Yingying Wu, Hongyu Chen, Ruiheng Yang, Dapeng Bao

**Affiliations:** ^1^Institute of Edible Fungi, Shanghai Academy of Agricultural Sciences, No. 1000 Jinqi Road, Fengxian District, Shanghai 201403, China; ^2^Marshall Institute for Interdisciplinary Research, Marshall University, Huntington, WV, USA

## Abstract

Fungal immunomodulatory proteins (FIPs) are a class of small proteins that have been extensively studied for their immunomodulatory activities. In this study, two novel FIPs from *Lentinus tigrinus* were identified and named Fip-lti1 and Fip-lti2. The bioactive characteristics of Fip-lti1 and Fip-lti2 were compared to a well-known FIP (LZ-8 from *Ganoderma lucidum*) to investigate the effect of Fip-lti1 and Fip-lti2 expression on concanavalin A- (Con A-) induced liver oxidative injury. Both Fip-lti1 and Fip-lti2 protected the livers from Con A-induced necrosis, as evidenced by decreased serum aminotransferase levels (AST, ALT) and relieved liver histology. Levels of proinflammatory cytokines (TNF-*α*, IL-1*β*, and IL-6) and oxidative stress (SOD, MDA) were shown to be reduced by expressing Fip-lti1 and Fip-lti2. In addition, the hepatoprotective effect of Fip-lti1, Fip-lti2, and LZ-8 correlated with ameliorating the imbalance of Th1/Th2 (IFN-*γ*/IL-4). The observed liver protection of Fip-lti1 and Fip-lti2 was mechanistically explored. Treatments with Fip-lti1 and Fip-lti2 regulated GATA3/T-bet expression, activated the decreased Nrf-2/HO-1 pathway, and countered the upregulated NLRP3/ASC/NF-*κ*Bp65 signaling in Con A-stimulated liver injury. Nrf2 activation was shown to be involved in the mechanisms underlying the protection of Fip-lti by RNA interference. In conclusion, we identified two new fungal proteins (Fip-lti1 and Fip-lti2) that can protect the liver from Con A-induced liver oxidative injury through the Nrf2/NF-*κ*B/NLRP3/IL-1*β* pathway.

## 1. Introduction

Many bioactive components have been identified from basidiomycete mushrooms; the identified molecules include polysaccharides [1], triterpenes [2], and fungal immunomodulatory proteins (FIPs) [3]. FIPs are a class of small proteins, which have shown anticancer, antiallergy, and antianaphylaxis activity and stimulation of immune cells to produce cytokines [4, 5]. Moreover, FIPs in pharmaceuticals or functioning as vaccine adjuvants can harness benefits of immune regulation [6, 7].

*Lentinus tigrinus* (*L. tigrinus*), a white-rot fungus that grows naturally on rotten hardwood during spring and summer in China [8], is an edible and medicinal mushroom containing a valuable combination of nutrients including high amino acid concentration and low sugar level [9]. Phenolics (one of the components isolated from this mushroom) reportedly have antioxidative properties [10]. However, no FIP genes have been isolated and characterized from *L. tigrinus* to date. Based on BLAST analysis, we identified a novel gene encoding fungal immunomodulatory protein in the *L. tigrinus* genome sequence (DOE Joint Genome Institute, https://www.jgi.doe.gov/) and designated it “Fip-lti.”

The liver, a vital organ for metabolism and detoxification, is continuously exposed to toxicants including chemical, biochemical, and biological insults [11]. Thus, the liver is at high risk for injury either directly or indirectly through the immune response. Con A is known to induce liver injury; hence, it has been utilized to generate the acute liver failure model. Con A activates T cells and natural killer T (NKT) cells, leading to hepatoinflammation or immune hepatitis. In a murine model, Con A-induced hepatic injury is featured with an abnormal immune response that mimics human T cell-mediated liver disease [12]. Elevated production of inflammatory cytokines—including tumor necrosis factor- (TNF-) *α*, interleukin- (IL-) 1*β*, and IL-6—was associated with the initiation of liver injury and rapid dysfunction [13]. Additionally, oxidative stress is closely related to inflammation as reactive oxygen species (ROS) are actively recruited leading to the cellular damage and progression of inflammatory disease [14].

So far, there have been no published systematic studies assessing the expressions and activities of FIPs under liver damage. Herein, we evaluated the protective effects of Fip-lti1 and Fip-lti2 in both in vitro and in vivo models. We further investigated the mechanisms of the hepatoprotective effect by Fip-lti1 and Fip-lti2 proteins and indicated the therapeutic potential of Fip-lti1 and Fip-lti2 for immunity-mediated liver injury.

## 2. Materials and Methods

### 2.1. Reagents

Con A and 3-(4,5-dimethylthiazol-2-yl)-2,5-diphenyltetrazolium bromide (MTT) were purchased from Sigma Chemical Company (St. Louis, MO, USA). IL-1*β*, IL-6, and TNF-*α* ELISA kits were provided by Nanjing KeyGen Biotech. Co. Ltd. (Nanjing, China). SOD and MDA kits were obtained from Nanjing Jiangcheng Bioengineering Institute (Nanjing, China). All antibodies were provided by Cell Signaling Technology (Danvers, MA, USA). The Lipofectamine® 3000 Reagent was obtained from Invitrogen (California, USA). The SuperFectin™ II siRNA transfection reagent was obtained from Pufei (Shanghai, China). siRNA of Nrf2 was purchased from Gene Pharma Co. (Shanghai, China).

### 2.2. Animals

Six-week-old male BALB/c mice (20 ± 2 g), supplied by Jiangning Qinglongshan Animal Cultivation Farm (Nanjing, China), were allowed to adapt to the animal facility for 1 week prior to experimentation. Animals were housed in an environmentally controlled room and given free access to food and water. All the animal experimental protocols were performed in accordance with the National Institutes of Health *Guide for the Care and Use of Laboratory Animals* [15] and were approved by our institute's ethics committee.

### 2.3. In Silico Analysis of Fip-lti1 and Fip-lti2

Fip-lti1 and Fip-lti2 were identified by a homology-based BLAST search using the amino acid sequence of LZ-8 in the *L. tigrinus* genome supplied by the DOE Joint Genome Institute (https://www.jgi.doe.gov/). Primary structure analyses of Fip-lti1 and Fip-lti2 were performed using the ProtParam and ProtScale web server [16] to confirm the details of the sequences, including molecular weight, theoretical isoelectric point (pI), each amino acid residue, and total numbers of negatively or positively charged residues of Fip-lti1 and Fip-lti2. The multiple sequence alignment was carried out using the ClustalW Program (https://www.ebi.ac.uk/Tools/msa/clustalw2/), and the aligned sequences were used to generate phylogenetic relationship using MEGA5 software [17]. Signal peptides and subcellular localizations were predicted by the SignalP (http://www.cbs.dtu.dk/services/SignalP/) [18] and TMHMM (http://www.cbs.dtu.dk/services/TMHMM-2.0/) [19] programs, respectively. The biologically significant sites of Fip-lti1 and Fip-lti2 were scanned using the PROSITE ExPASy proteomic tool (https://www.expasy.org/prosite/) [20]. MODELLER program was used for protein 3D modeling with crystal structures of LZ-8 (PDBID: 3F3H), FIP-fve (1OSY), and FIP-gmi (3KCW) as templates. The 3D structures were optimized by a 1000-step energy minimization with the steepest descent method and evaluated using PROCHECK [21].

### 2.4. Protein Expression and Purification

The core cDNA templates encoding the FIP-lti1/FIP-lti2/LZ-8—retrieved from the *L. tigrinus* (FIP-lti1/FIP-lti2) and *Ganoderma lucidum* (*G. lucidum*) (LZ-8) genomes—were synthesized by Sangon Biotech (Shanghai, China).

The synthesized products were cloned into the pUC57 vector and transformed into *Escherichia coli* (*E. coli*) DH5*α* competent cells using a standard protocol. The resultant construct was digested by the *BamHI* and *XhoI* enzymes at 37°C for 2 h. The released DNA fragments encoding the FIP-lti1/FIP-lti2/LZ-8 of *L. tigrinus* were cloned into the same enzyme-treated expression vector pET32a to generate plasmid pET32a-lti1/ pET32a-lti2/ pET32a-lz8 including a His-6 tag. The recombinant proteins were expressed in Rosetta (DE3) cells (Promega, Madison, WI, USA). The bacteria were cultured in Luria-Bertani liquid medium to a 0.3 optical density at 600 nm (OD600) at 37°C and then induced with 1 mM IPTG at 25°C for 4 h with shaking. The bacterial cells were then harvested and disrupted by ultrasonic disruption. The soluble fraction was separated by centrifugation at 15,000 rpm for 30 min at 4°C.

After centrifugation, the supernatant was loaded onto a Ni-NTA agarose column (2 ml) for purification. The bacteria were suspended in Buffer A (20 mM Tris, 300 mM NaCl, 10% glycerol, pH 8.0), and 2 mL Ni-NTA was added to the supernatant. The mixture was incubated at 4°C for 1 h and loaded onto an empty column and the elution was collected. Then, Buffer B (20 mM Tris, 300 mM NaCl, 10% glycerol, 20 mM imidazole, pH 8.0) was used to wash the column. Buffer C (20 mM Tris, 300 mM NaCl, 10% glycerol, 250 mM imidazole, pH 8.0) was used to elute and the eluent was collected. All the eluted samples were dialyzed into Buffer A (20 mM Tris, 300 mM NaCl, 10% glycerol, pH 8.0). The purified proteins were run on a 15% sodium dodecyl sulfate polyacrylamide gel electrophoresis (SDS-PAGE) and stained with Coomassie Brilliant Blue R-250. The purified proteins were removed the salt ions by dialysis and freeze-dried for standby application. Protein concentration was determined using the BCA Protein Assay Kit with bovine serum albumin as per the manufacturer's instructions.

### 2.5. Drug Administration

The five experimental groups comprised the (1) control group, (2) Con A group with Con A challenge, (3) Con A+Fip-lti1 (20 and 40 *μ*g/kg) group, (4) Con A+Fip-lti2 (20 and 40 *μ*g/kg) group, and (5) Con A+LZ-8 (20 and 40 *μ*g/kg) group. Protein LZ-8 served as a positive control. Protein Fip-lti1, Fip-lti2, and LZ-8 were dissolved in saline and intraperitoneally administered into the mice. Con A (15 mg/kg of body weight) was intravenously given 24 h after administration of Fip-lti1, Fip-lti2, or LZ-8 protein. Serum, liver, and spleen samples were collected 8 h after Con A injection.

### 2.6. Cell Culture and Treatment

Normal human liver cell L02 cell line was obtained from the Chinese Academy of Sciences (Shanghai, China). Cells were maintained in RPMI-1640 medium (Gibco, Grand Island, NY, USA) supplemented with 10% fetal bovine serum and 1% penicillin-streptomycin (Gibco) in a humidified incubator supplied with 5% CO_2_ at 37°C.

L02 cells were seeded into 96-well plates at a density of 5 × 10^4^ cells/ml in 100 *μ*l culture medium for 24 h. Then, cells were divided into five groups: the (1) vehicle group treated with PBS only, (2) Con A group treated with Con A (15 *μ*g/ml) dissolved in PBS, (3) Con A+Fip-lti1 (1, 10, and 100 pM) group, (4) Con A+Fip-lti2 (1, 10, and 100 pM) group, and (5) Con A+LZ-8 (1, 10, and 100 pM) group. The cells were incubated, respectively, with protein Fip-lti1, Fip-lti2, and LZ-8 at multiple concentrations for 1 h. In the meanwhile, the vehicle control cells were incubated with the same volume of PBS.

### 2.7. MTT Assay

Cell viability was assessed using MTT (Sigma). L02 cells were exposed to Fip-lti1, Fip-lti2, and LZ-8 (1, 10, and 100 pM) for 1 h followed by incubation with or without Con A for 24 h. Then, each well was added with 20 *μ*l of MTT (5 mg/ml) working solution and further incubated at 37°C for a further 4 h. Following the removal of culture medium, 150 *μ*l dimethyl sulfoxide (DMSO) was applied to dissolve the formazan crystals in each well. The absorbance values were detected at 450 nm with a microplate spectrophotometer (Tecan Group AG, Männedorf, Switzerland). Results were presented as percentage of average absorbance of control group MTT assay as per the manufacturer's instructions.

### 2.8. Serum Aminotransferase Levels

All the mice were sacrificed after the 8-hour Con A treatment. Blood was collected from the abdominal aorta. The serum was separated via centrifugation at 5000 rpm for 10 min at 4°C and stored at −80°C for further examination. Serum ALT and AST levels were determined with commercial kits supplied by Nanjing Jiancheng Bioengineering Institute. Briefly, 5 *μ*l of each sample was mixed with 20 *μ*l of matrix solution and kept mixing in a water bath of 37°C for 30 min, followed by adding 20 *μ*l of 2,4-dinitrophenylhydrazine and kept mixing in the water bath of 37°C for 30 min. Finally, 200 *μ*l of 0.4 mol/l sodium hydroxide was added and the reaction was read out at 510 nm after 15 min reaction.

### 2.9. Cytokine Assays

Serum or medium levels of IL-6, IL-1*β*, and TNF-*α* were detected with ELISA kits (KeyGen Biotech Co. Ltd., Nanjing, China). Absorbance was read at 450 nm by microplate spectrophotometer. Briefly, 100 *μ*l of samples at 1 : 2 dilution or different concentrations of the standards were pipetted into corresponding wells and incubated at room temperature for 120 min. After 5 washes, 100 *μ*l of biotin-conjugated antibody was added to each well (note: this biotin antibody has been pre-prepared without any further dilution), and incubated for 60 min at room temperature. After 5 washes, 100 *μ*l horseradish peroxidase-labeled streptavidin was added to each well and incubated for 20 min at room temperature. Finally, 100 *μ*l of chromogenic agent TMB solution was added and incubated for 20 min for color reaction, and then it was stopped with adding 50 *μ*l terminating solution. The plates were read at 450 nm.

### 2.10. Oxidative Stress Assay

Indicators of lipid peroxidation were used to investigate the antioxidative properties of Fip-lti1 and Fip-lti2 in the liver injury. Levels of MDA, serum SOD, and L02 cell supernatant were determined with the commercial kits as per the manufacturer's instructions (Jiangcheng Institute of Biotechnology, Nanjing, China).

Briefly, 20 *μ*l of each sample was mixed with 20 *μ*l of double distilled water and mixed homogenously in a 37°C water bath for 30 min, and then 20 *μ*l of enzyme working solution was added and kept mixing in the 37 °C water bath for 30 min. Add 20 *μ*l of enzyme diluent and then 200 *μ*l of substrate working solution to react for 15 min and the signals were read at 450 nm.

### 2.11. Flow Cytometry

T cells were isolated from the murine spleens and stained with fluorescence-conjugated antibodies (Abs). For Th1 and Th2 intracellular staining, the cells were prestimulated with cell stimulation cocktail at 37°C for 5 h and then stained with FITC-anti-CD4 for 30 min followed by fixation and permeabilization and finally exposed to PE-anti-IFN-*γ* and APC-anti-IL-4 antibodies for 1 h. For Treg staining, the cells were cultured with FITC-conjugated-CD4 and PE-conjugated-CD25 for 30 min followed by fixation and permeabilization and finally stained with APC-conjugated-Foxp3 antibodies for 1 h. All flow cytometric measurements were conducted using a FACSCalibur flow cytometer (BD Biosciences, San Jose, CA, USA).

### 2.12. Histopathological Evaluation

The livers were autopsied and fixed with 10% neutral formalin for 24 h. Briefly, the samples were dehydrated in graded alcohol, embedded in paraffin, and sectioned at 5 *μ*m thickness. Then, liver sections were stained with hematoxylin and eosin (H&E). Histology was evaluated by two pathologists using the histological scoring system [22].

### 2.13. Western Blot Analysis

Liver tissues and L02 cells were used to determine protein expression levels by Western blot. The samples were homogenized in lysis buffer (Sigma, St. Louis, MO, USA). After the homogenates were centrifuged at 12,000g for 15 min at 4°C, protein levels in the supernatants were determined using a bicinchoninic acid assay (BCA) kit (Beyotime). Proteins (40 *μ*g/lane) were separated in SDS-PAGE and transferred to polyvinylidene fluoride membranes (Millipore Corporation, Boston, MA, USA). The membranes were blocked for 2 h in 5% nonfat dry milk-TBS-0.1% Tween 20 for 2 h. The blots were then incubated with primary antibodies at 4°C overnight followed by a 2 h incubation with a horseradish peroxidase-conjugated secondary anti-rabbit antibody at room temperature and visualized by ECL KeyGen system (KeyGen Biotech. Co. Ltd.). Protein band density was scanned by a gel imaging system (ChemiScope 2850; Clinx Science Instruments Co. Ltd., Shanghai, China) and quantitatively analyzed (ChemiScope analysis program). Finally, the results were normalized to GAPDH in the samples.

### 2.14. Nrf2 siRNA Silencing

L02 cells in 6-well culture plates were grown to 70 % confluence for transfection with Lipofectamine™ 3000 as per the manufacturer's instructions. Specific siRNA for Nrf2 a (GGGUAAGUCGAGAAGUGUUTT) and b (AACACUUCUCGACUUACCCTT) isoforms and scrambled control siRNAs were designed by Gene Pharma Co. (Shanghai, China). Five microliters of siRNA, 5 *μ*l Lipofectamine™ 3000, and 95 *μ*l serum-free cultural medium (Opti-MEM, Invitrogen) were mixed and incubated at room temperature for 20 min. Then, 800 *μ*l of Opti-MEM medium was dropwise added to each well. The siRNA mixture was then added to the L02 cells. The siRNA transfection medium was replaced 6 hours later, and cells were then further incubated for 24 h before exposed to Fip-lti1, Fip-lti2, and Con A. The knockdown efficiency was validated by analysis of myocardial injury markers and Western blotting. Then, transfected cells were divided into 8 groups: (a) control, (b) Con A-induced L02 cells, (c) Con A+Fip-lti1 (100 pM) group, (d) Co nA-induced siRNANrf-2 L02 cells+Fip-lti1 (100 pM) group, (e) Con A+Fip-lti2 (100 pM) group, (f) Con A-induced siRNANrf-2 L02 cells+Fip-lti2 (100 pM) group, (g) Con A+LZ-8 (100 pM) group, and (h) Con A-induced siRNANrf-2 L02 cells+LZ-8 (100 pM) group.

### 2.15. Nrf-2 Activation Experiment

L02 cells were grown to 70% confluence. Then, cells were divided into 8 groups: (a) control, (b) Fip-lti1 (100 pM), (c) Fip-lti2 (100 pM), (d) LZ-8 (100 pM), (e) BW1263W94 (Nrf-2 agonist, 100 pM), (f) Fip-lti1 (100 pM)+BW1263W94 (100 pM), (g) Fip-lti2 (100 pM)+BW1263W94 (100 pM), and (h) LZ-8 (100 pM)+BW1263W94 (100 pM). The cells were then treated as indicated for each group for 24 hours and were then collected to detect the expression of Nrf-2 and HO-1 proteins.

### 2.16. Quantitative Real-Time RT-PCR Analysis

L02 cells were seeded into six-well culture dishes and treated with substances as indicated. Thereafter, the cells were lysed and total RNA was isolated using a commercial kit, and quantitative PCR (qPCR) was performed as described previously. The primer sequences are listed below: HO-1: F: 5′-TGTATCCGCTATGGTTACAC-3′, R: 5′-GGTGGCACTGGCAATGTTGG-3′ and GAPDH: F: 5′-GTCATCCATGACAACTTTGG-3′, R: 5′-GAGCTTGACAAAGTGGTCGT-3′.

### 2.17. Statistical Analysis

Quantitative variables are presented as mean values ± SDs. Differences between groups were analyzed by one-way analysis of variance (ANOVA) with GraphPad Prism. *P* < 0.05 was considered statistically significant.

## 3. Results

### 3.1. Two Novel FIPs: Fip-lti1 and Fip-lti2

The nucleotide sequence that encodes for Fip-lti1 and Fip-lti2 consists of 339 and 345 base pairs (bp) or 113 and 115 amino acids (aa), respectively. The calculated molecular weights are 12.61 kDa and 12.80 kDa. The peptide sequence of Fip-lti1 consists of 13 strongly basic (+) amino acids (Arg, Lys) and 11 strongly acidic (−) amino acids (Asp, Glu), resulting in an 8.66 pI value (alkaline). In contrast, Fip-lti2 consists of 10 strongly basic (+) amino acids (Arg, Lys) and 12 strongly acidic (−) amino acids (Asp, Glu), leading to a 5.49 pI value (acidic). Both Fip-lti1 and Fip-lti2 do not carry signal peptide and transmembrane helices.

**Figure 1:**
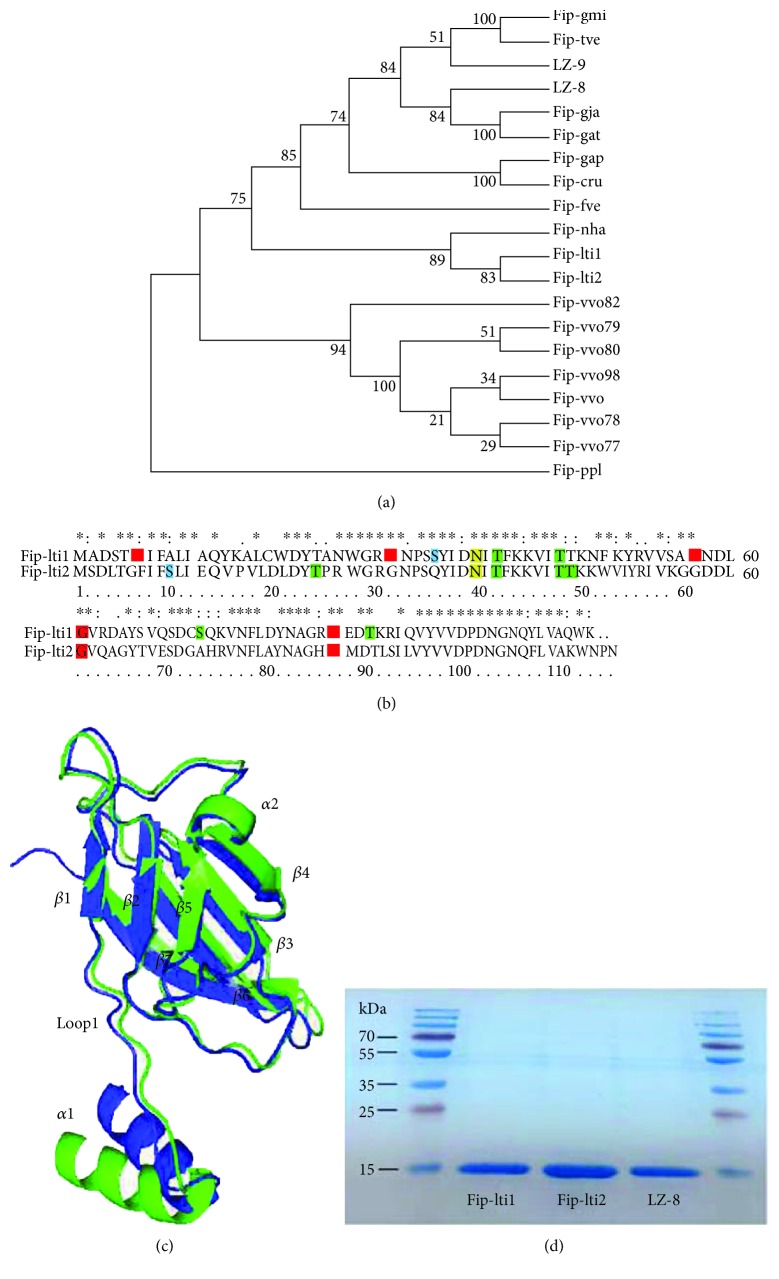
Phylogenetic and structural features and expression of Fip-lti1 and Fip-lti2. (a) Phylogenetic analysis of FIPs. (b) Sequence alignment of Fip-lti1 and Fip-lti2. Specific posttranslational modification sites are indicated as colored amino acids: myristoylation (red), casein kinase II phosphorylation (blue), protein kinase C phosphorylation (green), and N-glycosylation (yellow). (c) Superposition of the main chain backbone of Fip-lti1 (green) with Fip-lti2 (blue). (d) SDS-PAGE analysis of purified FIPs.

As shown in Figure 1(a), the phylogenetic relationship among the identified FIPs reveals that Fip-lti1 and Fip-lti2 form a unique separate branch, indicating a substantial divergence from all FIPs, but Fip-nha. Amino acid modification feature scan suggested that Fip-lti1 contains five putative N-myristoylation sites, four putative protein kinase C phosphorylation sites, one casein kinase II phosphorylation site, and one putative N-glycosylation site; G61 and G86 of putative N-myristoylation sites, T39 and T45 of putative protein kinase C phosphorylation sites, and one putative N-glycosylation site are shared by both Fip-lti1 and Fip-lti2 (Figure 1(b)).

Crystal structures of LZ-8, Fip-fve, and Fip-gmi were used as templates to build the homology model of Fip-lti1/Fip-lti2, which was further refined by energy minimization. The predicted 3D structure of Fip-lti1/Fip-lti2 shows no residue in disallowed region of the Ramachandran plot. As shown in Figure 1(c), the predicted 3D structure of Fip-lti1/Fip-lti2 resembles the FIP typical structure with a fibronectin type III (FNIII) folder, which represents a transition between the seven *β*-stranded s-type and the eight *β*-stranded h-type topologies. The predicted structures of Fip-lti1 and Fip-lti2 can be superimposed except for the N-terminal *α*-helix, loop1, *β*1, and *β*2 regions. The N-terminal *α*-helix and loop1 regions could affect the formation of homodimer and performance of FIP [23].

The DNA fragments encoding FIPs of *L. tigrinus* were cloned and FIPs were expressed as a His-fusion protein in *E. coli*. The two FIPs of *L. tigrinus* migrated at the same position as LZ-8, a known 13 kDa protein in Tris-glycine SDS-PAGE, as suggested by the theoretical calculations (Figure 1(d)). And the lipopolysaccharides (LPS) had been removed, and the concentration of LPS was less than 5 EU/*μ*g proteins (data not shown). According to Xu's report, LPS at this concentration in the culture media has obvious effects on neither the growth nor morphology of the cell [24].

### 3.2. Effects of Fip-lti1 and Fip-lti2 on Hepatic Necrosis

**Figure 2:**
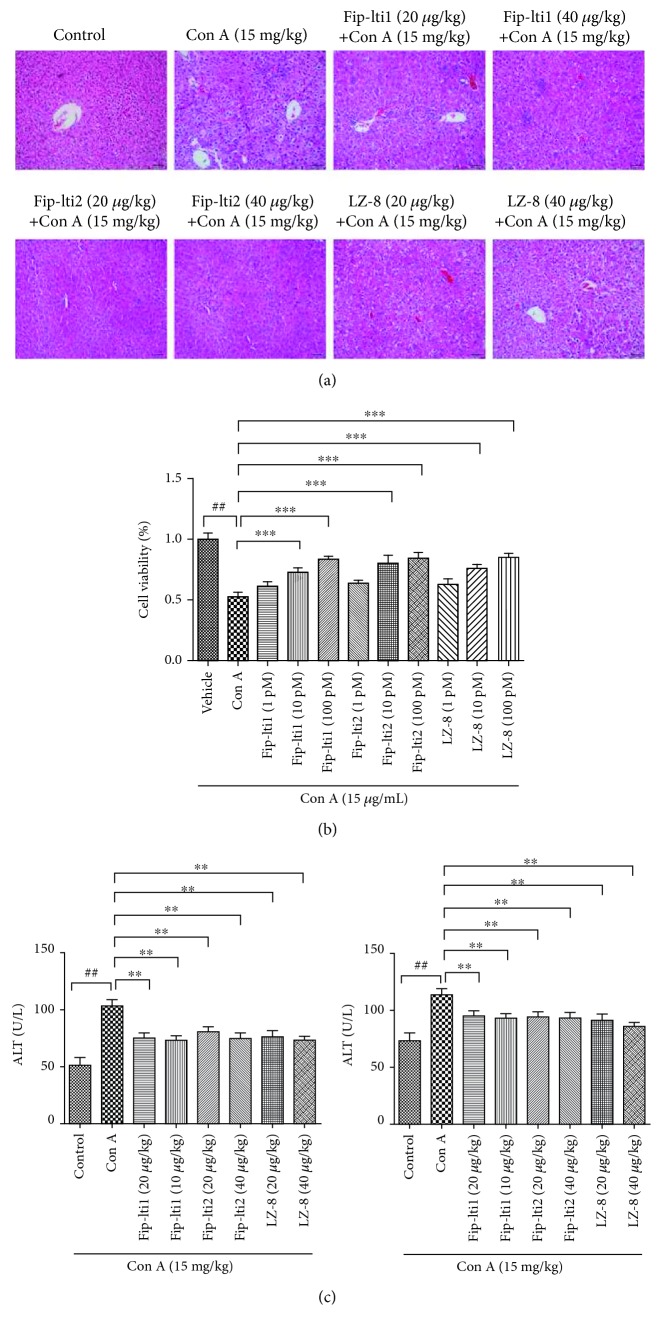
Protection of Con A-induced liver injury by Fip-lti1 and Fip-lti2. (a) Liver histology of different groups and sections was stained by H&E. (b) Differences in L02 cell viability among different groups. (c) Serum AST and ALT levels among different groups. Each experiment was repeated at least three times and a representative one is shown. ## and ∗∗ indicate *P* < 0.01; ### and ∗∗∗ indicate *P* < 0.001.

Protective function of Fip-lti1 and Fip-lti2 in mice with Con A-induced hepatic necrosis was assessed by histology (Figure 2(a)). Liver sections from the control group showed normal liver architecture and cell morphology. Con A administration led to remarkable histological changes including extensive vacuolization and necrosis with inflammatory cell infiltration in vast areas. However, the pretreatment with Fip-lti1 or Fip-lti2 at 20 and 40 *μ*g/kg mitigated liver injury to some extent. To investigate protective concentrations of Fip-lti1 and Fip-lti2, an *in vitro* experiment with L02 cells that were analyzed by MTT assay was performed. After Con A was included, the viability of L02 cells was significantly lower compared with the control group. However, treatments with different concentrations of Fip-lti1 or Fip-lti2 (1, 10, and 100 pM) effectively stabilized the cell viability in a dose-dependent manner (Figure 2(b)).

Serum AST and ALT levels were determined to assess extents of acute liver injury. Both serum AST and ALT levels in the control group remained normal. Administration of Con A in mice significantly resulted in higher levels of both AST and ALT compared with the control group. However, both ALT and AST levels in mice pretreated with Fip-lti1 or Fip-lti2 were significantly lower than those in mice without treatment after Con A administration (Figure 2(c)).

### 3.3. Effect of Fip-lti1 and Fip-lti2 on the Regulation of T cells

**Figure 3:**
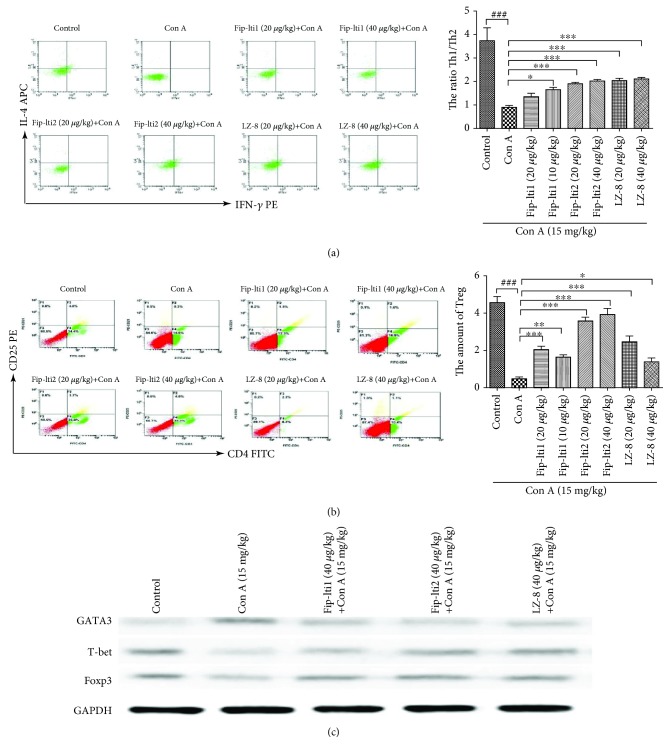
Fip-lti1, Fip-lti2, and LZ-8 mitigated the liver injury in Con A mice by regulating T cells. (a) Fip-lti1, Fip-lti2, and LZ-8 restored the balance between Th1 and Th2 cells in the murine spleens. (b) Fip-lti1, Fip-lti2, and LZ-8 upregulated Treg cells in the spleens. (c) T cell-related protein expression levels in the livers. Each experiment was repeated at least three times and a representative one is shown. ∗ indicates *P* < 0.05, ∗∗ indicates *P* < 0.01, and ### and ∗∗∗ indicate *P* < 0.001.

To determine how the liver protective function of Fip-lti1 and Fip-lti2 was mediated, we investigated fractions of Th1, Th2, and Treg cells in the murine spleens. A lower ratio of IFN-*γ*/IL-4 in the Con A-treated mice was seen compared to the normal mice, and there were a significantly low number of CD4^+^CD25^+^ T cells. However, the number of CD4^+^CD25^+^ T cells and the ratio of IFN-*γ*/IL-4 in mice treated with Fip-lti1, Fip-lti2, or LZ-8 were significantly higher compared to no FIP treatment (Figures 3(a) and 3(b)). Consistent with that, the Western blot analysis of Th1, Th2, and Treg-specific protein in liver tissues showed that Con A treatment decreased the expression of T-bet and Foxp3 and increased GATA3. However, pretreatment of Fip-lti1 and Fip-lti2 blocked the function of Con A (Figure 3(c)).

### 3.4. Effects of Fip-lti1 and Fip-lti2 on Antioxidative Defense

**Figure 4:**
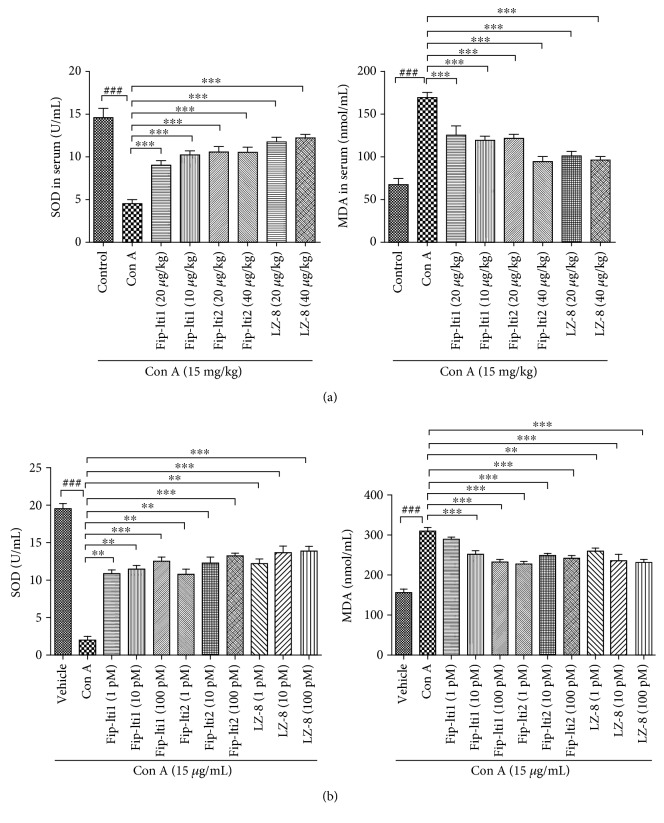
Fip-lti1 and Fip-lti2 retarded the changes in serum/medium levels of SOD and MDA induced by Con A. (a) Serum SOD and MDA levels. (b) Medium SOD and MDA levels in L02 cells. Each experiment was repeated at least three times and a representative one is shown. ∗ indicates *P* < 0.05, ∗∗ indicates *P* < 0.01, and ### and ∗∗∗ indicate *P* < 0.001.

The expressed SOD and MDA activity in both *in vivo* and *in vitro* experiments was assayed to determine the biologic function of Fip-lti1 and Fip-lti2 on oxidative stress. Con A administration significantly downregulated SOD activity and upregulated MDA level in serum compared to the control group. Conversely, the treatments with Fip-lti1, Fip-lti2, or LZ-8 (20 and 40 *μ*g/kg) significantly blunted the extent of changes in the serum levels of SOD and MDA. Similarly, the oxidative stress reached a higher level in L02 cell supernatant in response to Con A exposure compared to the control group. Notably, the treatments with Fip-lti1 or Fip-lti2 (1, 10, and 100 pM) retarded changes in the levels of SOD and MDA (Figures 4(a) and 4(b)).

### 3.5. Effects of Fip-lti1 and Fip-lti2 on Inflammatory Pathway

**Figure 5:**
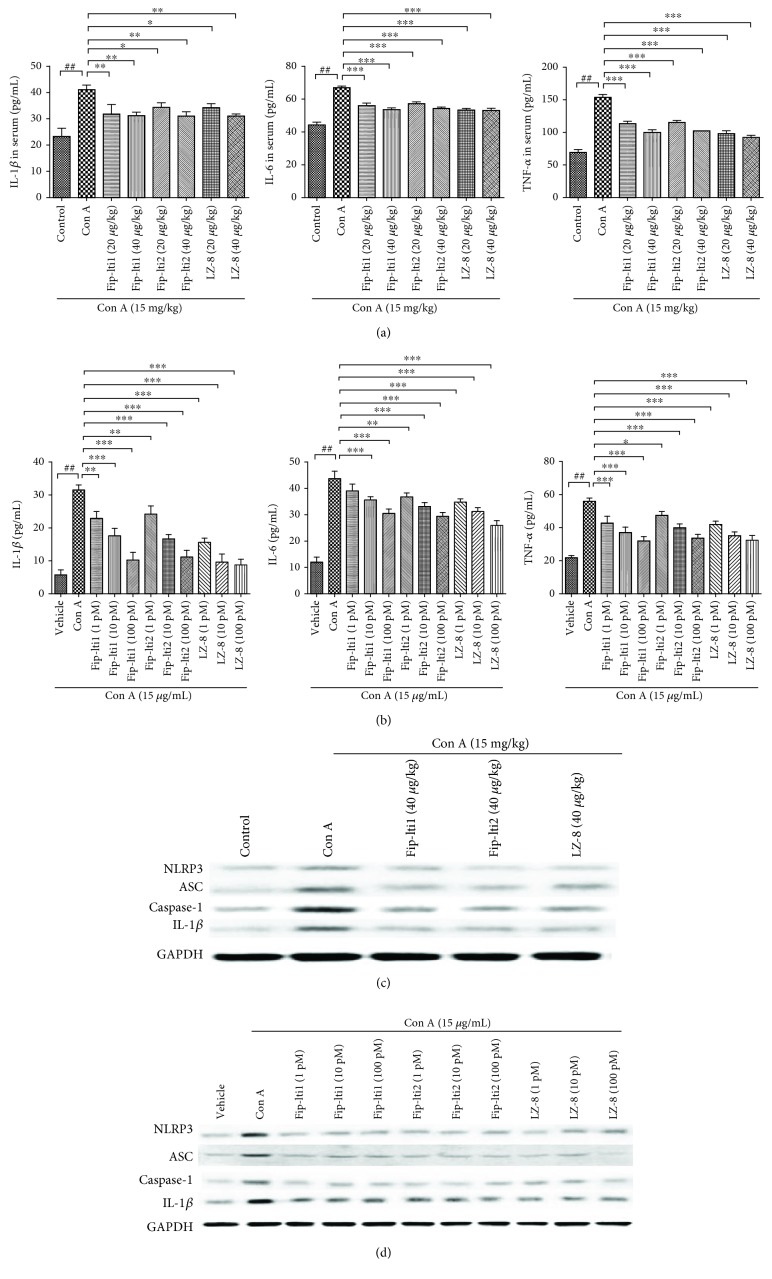
Levels of proinflammatory cytokines were lower with the treatment with Fip-lti1 or Fip-lti2. (a) Serum IL-1*β*, IL-6, and TNF-*α* levels. (b) IL-1*β*, IL-6, and TNF-*α* levels in L02 medium. (c, d) Inflammation-associated protein expression levels in the livers and in L02 cells. Each experiment was repeated at least three times and a representative one is shown. ∗ indicates *P* < 0.05, ∗∗ indicates *P* < 0.01, and ### and ∗∗∗ indicate *P* < 0.001.

Proinflammatory cytokines IL-6, IL-1*β*, and TNF-*α* were measured using ELISA kits to determine the *in vivo* and *in vitro* inflammatory responses. Following exposure to Con A, higher serum IL-1*β*, IL-6, and TNF-*α* levels were detected compared to the control group. However, an inclusion of Fip-lti1, Fip-lti2, or LZ-8 (20 and 40 *μ*g/kg) led to lower serum levels of IL-1*β*, IL-6, and TNF-*α*. Similar effects of Fip-lti1, Fip-lti2, or LZ-8 were also observed in L02 cells following the exposure to Con A (Figures 5(a) and 5(b)).

NLRP3 is the most definitive inflammasome that consists of NLRP3, ASC, and caspase-1. After exogenous stimulation, NLRP3 inflammatory corpuscles are activated to form the NLRP3/caspase-1 signaling pathway. As shown in Figures 5(c) and 5(d), Con A treatment increased the expression of NLRP3, ASC, caspase-1, and the downstream IL-1*β* both in liver tissues and in L02 cells. Moreover, the regulation of protein expression was all restored by Fip-lti1 and Fip-lti2.

### 3.6. Effect of Fip-lti1 and Fip-lti2 on Expressing Components of the Signaling Pathway

**Figure 6:**
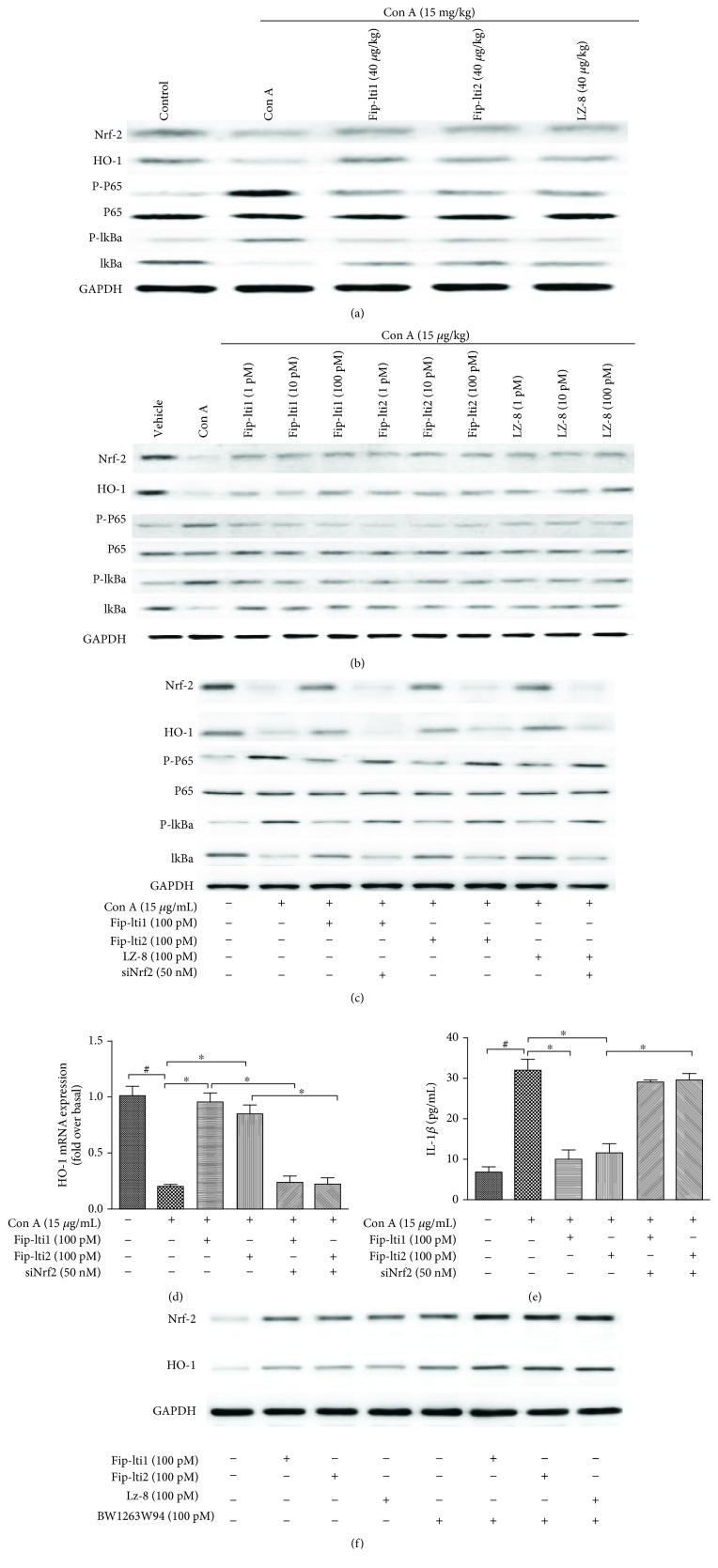
Effects of Fip-lti1 and Fip-lti2 on the protein expression levels of the signaling pathway in the livers (a) in L02 cells (b). (c) Nrf2 expression on the effect of the NF-*κ*B pathway of Fip-lti1 and Fip-lti2 (d) one-step real-time PCR assays to determine the mRNA of HO-1. (e) IL-1*β* secretion was determined in response to Nrf2 siRNA by ELISA. (f) Nrf2 activation by Fip-lti1, Fip-lti2, and its agonist BW1263W94. Each experiment was repeated at least three times and a representative one is shown. ∗ indicates *P* < 0.05, ∗∗ indicates *P* < 0.01, and ### and ∗∗∗ indicate *P* < 0.001.

It was proposed that immunity, oxidation, and inflammation are involved in the pathogenesis of liver injury induced by Con A. Protein levels of components in the signaling pathway were determined with Western blot. As shown in Figures 6(a) and 6(b), the expression levels of p-NF-*κ*Bp65 and p-I*κ*B*α* were higher while levels of Nrf-2 and HO-1 were lower in the livers exposed to Con A. Pretreatments with Fip-lti1, Fip-lti2, or LZ-8 (40 *μ*g/kg) effectively retarded the changes in the expression of these proteins in the livers. Additionally, the results from L02 cells suggested that Fip-lti1 and Fip-lti2 pretreatments retarded the decreased Nrf-2/HO-1 pathway as well as the upregulated NF-*κ*Bp65 signaling in Con A-stimulated L02 cells.

Silencing Nrf2 with siRNA was used to confirm the potential effect of Nrf2 on the protection of Fip-lti in Con A-induced dysfunction. As shown in Figure 6(c), Nrf2 knockdown mitigated the effects of Fip-lti1, Fip-lti2, or LZ-8 on the NF-*κ*B pathway, showing that Nrf-2 is required for the functions of Fip-lti1, Fip-lti2, or LZ-8 in L02 cells. And in Figures 6(d) and 6(e), Nrf2 knockdown inhibited the restoration of the expression of antioxidant enzymes HO-1 by Fip-lti and abolished the effect of Fip-lti in IL-1*β* secretion.

In Nrf-2 activation experiment (Figure 6(f)), we found that Fip-lti1, Fip-lti2, or LZ-8 significantly increased Nrf-2 and HO-1 protein expression in normal L0 cells. BW1263W94 (Nrf-2 agonist) also significantly increased Nrf-2 and HO-1 protein expression. Next, BW1263W94 and Fip-lti1, Fip-lti2, or LZ-8 were added to normal L02 cells at the same time. We found that Fip-lti1, Fip-lti2, or LZ-8 can increase the expression of Nrf-2 and HO-2 in conjunction with BW1263W94. This result shows that Fip-lti1, Fip-lti2, or LZ-8 can effectively activate the expression of Nrf-2 and HO-1.

## 4. Discussion

Fungal immunomodulatory protein, isolated from higher basidiomycetes, is a class of small molecule protein with immunomodulatory activity. Purification of FIPs from wild or cultivated mushrooms remains low yielded, complicated, time-consuming, and costly. Such effort is now focused on identifying new FIPs by other molecular methods and characterizing their expression. To obtain sufficient FIPs for practical applications, many genetic engineering approaches have been performed to improve the yields of FIPs. So far, the most effective system to express new FIPs is DNA recombination. However, the expression systems are faced with the disadvantages of long growth cycle, high cost, and complicated purification procedure. In this article, for the first time, we tried to isolate new FIP genes from genomic DNA data and express them to obtain Fip-lti1 and Fip-lti2. This bioinformatics approach is time-saving and efficient. Furthermore, we identified the immunomodulatory activity of Fip-lti1 and Fip-lti2.

In this study, normal human hepatic L02 cells and mice were utilized as models to investigate the protective function of Fip-lti1 and Fip-lti2 and the potential molecular mechanisms in Con A-induced liver injury. We demonstrated (*in vitro and in vivo*) that Fip-lti1 and Fip-lti2 protected liver cells against Con A-induced injury. Both Fip-lti1 and Fip-lti2 can retard proinflammation cytokine production and exert antioxidative activity. Autoimmune hepatitis is a liver inflammatory disease with increasing incidence. No effective treatment is available because of a lack of mechanistic understanding of this disease [25]. Con A-induced liver injury is a well-established model to investigate mechanisms of immunity-associated liver diseases [26] and we used this model to elucidate the beneficial functions of Fip-lti1 and Fip-lti2.

Furthermore, we showed that the protective effect of Fip-lti1 and Fip-lti2 was likely mediated through modulating the GATA3/T-bet pathway-mediated restoration of Th1/Th2 balance. CD4^+^ Th cells consist of three populations; each demonstrates a distinctive pattern of major cytokine production: IFN-*γ*-producing CD4^+^ T cells (Th1), IL-4-producing CD4^+^ T cells (Th2), and IL-17-producing CD4^+^ T cells (Th17). Since the discovery of the importance of Th1-Th2 balance in mice in 1986, many studies have shown how imbalanced Th1-Th2 functions may upset immune homeostasis and cause liver injury [27]. We found that the hepatoprotective effect of Fip-lti1, Fip-lti2, and LZ-8 similarly correlated with ameliorating the imbalanced Th1/Th2 (IFN-*γ*/IL-4) function, which was likely mediated through modulating the expression of GATA3 and T-bet in the spleen. In addition, the loss of functional Tregs was attributed to the abnormal inflammatory and immune response detected in liver injury and sufficient expression of cell surface markers CD4 and CD25 and the transcription factor FoxP3 is indispensable for Tregs functions [28]. We showed that a supplement of regulatory T cells contributed to the recovery of liver Con A-induced injury.

The immune response process can facilitate the oxidative and inflammatory reaction [29]. MDA is a reliable index reflecting the extent of cell injury. SOD indicates a cellular capacity to scavenge excessive ROS. It has been suggested that an imbalanced SOD and MDA level was involved in the pathogenesis of liver injury [30]. Inflammatory cytokines including TNF-*α*, IL-1*β*, and IL-6 are involved in initiating and aggravating the inflammation. Our results showed that the levels of TNF-*α*, IL-1*β*, IL-6, and MDA were significantly higher, and the SOD activity was lower (both *in vivo* and *in vitro*), with Con A treatment compared to the control group. However, such proinflammatory cytokine production and oxidative markers were effectively retarded by administering Fip-lti1, Fip-lti2, or LZ-8. The results suggest that Fip-lti1 and Fip-lti2 possess anti-inflammatory and antioxidative properties as demonstrated in Con A-induced liver injury.

The results were shown that Fip-lti1 and Fip-lti2 could effectively regulate the level of IL-1*β*. In order to understand the role of NLRP3 inflammatory complexes in the Fip-lti1- and Fip-lti2-decreased IL-1*β* production, the expression of NLRP3, ASC, and caspase-1 of inflammatory body components was analyzed, and the results showed that Fip-lti1 and Fip-lti2 could inhibit the expression of NLRP3, ASC, and caspase-1 induced by Con A.

Earlier studies suggested that NF-*κ*B was critical in the liver injury [31]. NF-*κ*B is activated by various stimuli [32]. Clearly, NF-*κ*B initiates and regulates the inflammatory process and is responsible for the generation of proinflammatory cytokines. As an inhibitor of NF-*κ*Bp65, I*κ*B*α* is governed by I*κ*B kinases (IKKs) [33]. IKK complex consists of IKK-*α* and IKK-*β*. NF-*κ*B subunit p65 is activated via the phosphorylation and degradation of the I*κ*B*α*. Our results showed that the levels of p-I*κ*B*α* and p-NF-*κ*Bp65 in Con A-induced livers and L02 cells were significantly lower following Fip-lti1 or Fip-lti2 treatment, suggesting the anti-inflammatory properties of Fip-lti1 and Fip-lti2 are mediated through regulating the NF-*κ*B pathway. Activation of the NF-*κ*B signaling pathway only induced the production of pro-IL-1*β*, and the maturation of IL-1b requires the activation of NLRP3 inflammasomes and further cleavage by caspase-1.

**Figure 7:**
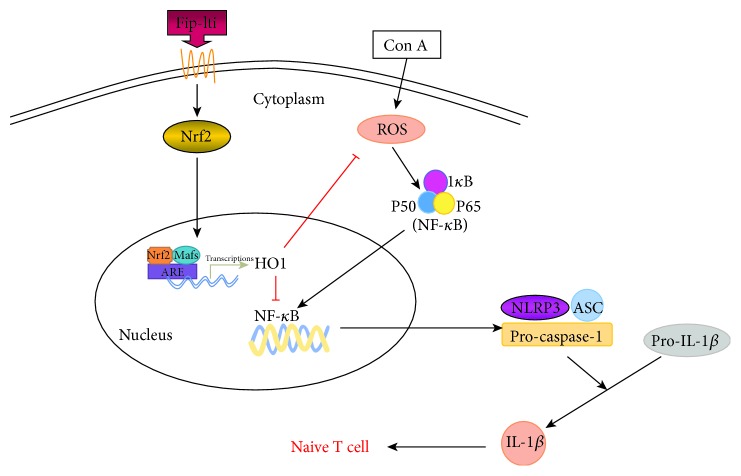
Illustration for the mechanism underlying Fip-lti1 and Fip-lti2 for the improvement of concanavalin A-induced liver injury. Fip-lti1 and Fip-lti2 ameliorate Con A-induced liver injury by the Nrf2-mediated NF-*κ*B signaling and NLRP3 inflammasome inactivation.

In response to oxidative stress, cells upregulate transcription factor nuclear factor erythroid 2-related factor 2 (Nrf2). Nrf2 translocates into the nucleus and binds to antioxidant response element (ARE) and regulates heme oxygenase-1 (HO-1) [34]. HO-1 can regulate the oxidative reaction and control ROS levels. In this study, both Nrf2 and HO-1 expressions were downregulated in the Con A-treated group. Such downregulations can be blunted with the Fip-lti1, Fip-lti2, or LZ-8 treatment, suggesting that Fip-lti1 and Fip-lti2 might relieve the oxidative stress through the Nrf2/HO-1 pathway. We hypothesized that increased Nrf2 activation by Fip-lti1 and Fip-lti2 will prevent NF-*κ*B activation and result in lower inflammation in Con A-challenged cells. And the result has shown that siRNA of Nrf2 prevented the secretion of IL-1*β*, which was activated by the NF-*κ*B/NLRP3 pathway; in addition, it mitigated the effect on the NF-*κ*B pathway. The differentiation of T helper (Th) cells is critically dependent on cytokine milieu. The innate immune monocytes produce IL-1*β* which can affect the development of Th cells. A possible mechanism of action of Fip-lti is illustrated in Figure 7.

This study mainly focused on investigating the protective effect of Fip-lti1 and Fip-lti2 in hepatic injury models and the underling pathway. We note the differences in both *β*1and *β*2 regions between Fip-lti1 and Fip-lti2. Both *β*1and *β*2 regions are located in the FNIII fold, which is considered important for different immunomodulatory functions [35]. Our structural comparative analysis indicates that Fip-lti1 and Fip-lti2 may possess different immunomodulatory functions, which we will elucidate in our future experiments.

In conclusion, Fip-lti1 and Fip-lti2 effectively attenuated the Con A-induced liver oxidative injury *in vivo* and *in vitro* via the Nrf2/NF-*κ*B/NLRP3/IL-1*β*-mediated pathway, which restored the Th1/Th2 balance. Further studies are warranted to investigate the effects of Fip-lti1 and Fip-lti2 in clinical applications.

## Data Availability

All the data used to support the findings of this study are included in the article.
